# Cardiovascular Manifestations and Interventions in Children Living in a Histoplasmosis Endemic Region

**DOI:** 10.1007/s00246-025-04123-w

**Published:** 2025-12-02

**Authors:** Cassandra Campbell, Sandra Arnold, Mario Briceno-Medina, Michael Perez, Shyam Sathanandam

**Affiliations:** 1 Pediatric Cardiology, Children’s Hospital of Pittsburgh, University of Pittsburgh Medical Center, Pittsburgh, PA USA; 2 Pediatric Infectious Disease, Le Bonheur Children’s Hospital, University of Tennessee Health Science Center, Memphis, TN USA; 3 Pediatric Cardiology, Le Bonheur Children’s Hospital, University of Tennessee Health Science Center, Memphis, TN USA; 4 Pediatric Cardiology, Texas Center for Pediatric and Congenital Heart Disease, Dell Children’s Medical Center, Austin, TX USA; 5 Pediatric Cardiology, Nicklaus Children’s Hospital, Miami, FL USA

**Keywords:** Histoplasmosis, Pediatrics, Cardiac histoplasmosis, Interventional cardiology

## Abstract

Histoplasmosis in children remains understudied, drawing most clinical understanding from adult populations. Located in a histoplasmosis-hyperendemic region, our institution aimed to characterize the cardiovascular manifestations and interventional treatments in children diagnosed with histoplasmosis. We conducted a retrospective cohort study of 160 patients under 18 years of age diagnosed with histoplasmosis between January 2009 and March 2023 at a tertiary-care children’s hospital. Data reviewed included clinical presentation, imaging, electrocardiograms, echocardiograms, and catheter-based or surgical interventions. Of the 160 patients, 65 (41%) demonstrated cardiovascular involvement from mediastinal manifestations of histoplasmosis (mediastinal lymphadenopathy, mediastinal granuloma, and fibrosing mediastinitis) including all 8 patients with fibrosing mediastinitis. Among these 65 children, pericarditis with pericardial effusion was the most common (58%), 30 patients requiring urgent intervention. Other manifestations from mass effect included pulmonary artery stenosis (34%), superior vena cava (SVC) stenosis (15%), pulmonary vein stenosis (8%), inferior vena cava (IVC) stenosis (3%), and aortic arch obstruction (2%). Fourteen patients underwent catheter-based stenting procedures with a total of 22 stents placed (17 in pulmonary artery branches, 4 in pulmonary veins, and 1 in the SVC), typically not performed during the initial presentation. Five patients underwent mass resection for cardiac indications. Cardiovascular complications from histoplasmosis-related mediastinal lymphadenopathy in children are significant, presenting with pericarditis or compression of central vascular structures. Fibrosing mediastinitis was consistently associated with cardiac involvement, frequently necessitating intervention. Surgical repair is challenging due to extensive fibrosis. However, percutaneous vascular interventions are feasible and effective in managing vascular obstructions. Early recognition and timely intervention are critical to prevent irreversible vascular atresia.

**Supplementary Information:**

The online version contains supplementary material available at https://doi.org/10.1007/s00246-025-04123-w.

## Introduction

*Histoplasma capsulatum* is a dimorphic fungus endemic to the United States with regions of hyperendemnicity in the Ohio and Mississippi River valleys, thriving in these regions due to their moderate climate and humidity [1]. Inhalation of airborne spores is the primary route of entry into the human body, leading to infection that can range from localized pulmonary mediastinal lymphadenopathy and pulmonary nodules to pneumonia or disseminated infection, the latter predominantly seen in immunocompromised hosts [2]. While many individuals have subclinical infection or experience only mild respiratory symptoms, some may develop complications related to mass effect from or inflammation of mediastinal lymph nodes [3].

Histoplasmosis in children has been less extensively studied than adults. Notably, a retrospective cohort study of 73 children and a systematic review of 83 pediatric cases have provided insights into patient characteristics, clinical manifestations, diagnosis, treatment, and outcomes [4, 5]. However, these studies did not focus on cardiac involvement. One study, from Columbus, Ohio, discussed pericarditis resulting from histoplasmosis in 18 children and therapeutic cardiac procedures, but broader investigations into other cardiac manifestations in children are scarce [6].​

In adults, histoplasmosis is known to affect the heart, presenting as pericarditis with or without pericardial effusion, endocarditis affecting native and prosthetic valves, mycotic aortic aneurysm, aortic graft infection, and myocarditis [7–11].​ Additional chronic changes are constrictive pericarditis, pulmonary hypertension, and extrinsic compression of the heart or major vessels can occur due to complications arising from mediastinal lymph node involvement [12, 13].

Mediastinal lymphadenopathy is a common clinical manifestation of histoplasmosis in children, which is frequently subclinical and detected incidentally on chest imaging done for a variety of symptoms. Lymph nodes can regress with time but can also progress to two distinct pathologies: mediastinal granuloma and fibrosing mediastinitis. Mediastinal granuloma occurs when a large number of infected mediastinal, hilar, or subcarinal lymph nodes coalesce and form a large caseating mass with a fibrous capsule. In contrast, fibrosing mediastinitis results from an uncontrolled fibrotic reaction to the infection, characterized by mature collagen deposition upon biopsy. Both conditions can progress slowly over years, potentially causing mass effect or obstruction of chest structures, including the heart and major vessels, bronchi, trachea, esophagus, thoracic duct, and recurrent laryngeal nerve. Mediastinal granuloma typically follows a more benign course although spontaneous drainage of necrotic material into the mediastinum can result in sudden, severe illness [14].​

Fibrosing mediastinitis arises from a variety of etiologies, adult studies have reported high mortality rates (20–40%) associated with debulking and reconstructive surgeries. Consequently, percutaneous angioplasty and/or stent placement are often preferred for managing vascular stenosis in adults, given the challenges of surgical dissection due to tissue destruction and the limited role of antifungal agents and corticosteroids at this stage of disease process of histoplasmosis [2, 15, 16]. In pediatric cases, such interventions have been documented but are limited to single case reports [17].​

The primary aim of this study is to describe the cardiac complications of mediastinal histoplasmosis including mediastinal lymphadenopathy, mediastinal granuloma, and fibrosing mediastinitis in children presenting to Le Bonheur Children’s Hospital, in Memphis TN, which is situated in a hyperendemic region for histoplasmosis. We hypothesized that among children diagnosed with histoplasmosis at our institution, chest pain secondary to pericarditis with pericardial effusion is the most common cardiac manifestation. Our secondary aim is to describe the natural history and interventions of mediastinal cardiovascular complications of histoplasmosis in children.

## Methods

This retrospective cohort study included children under 18 years of age who were admitted to Le Bonheur Children’s Hospital, in Memphis, TN for the diagnosis of histoplasmosis between January 2009 and March 2023. This study included patients’ initial presentation that led to the diagnosis as well as subsequent visits for their histoplasmosis diagnosis that occurred in the time frame of the study. Institutional Review Board approval with a waiver of consent was obtained for this study. Patients were identified using ICD-9 and ICD-10 codes for histoplasmosis and its intrathoracic complications (see Supplemental Materials).

### Diagnostic Criteria

Diagnosis was established based on (1) the presence of a compatible clinical syndrome with mediastinal lymphadenopathy or mass on chest CT and (2) absence of clinical suspicion for tuberculosis (negative exposure history, negative skin testing or interferon gamma release assay) or lymphoma (history and mass characteristics) [18, 19]. Patients with definite histoplasmosis had 1 and 2 above with histopathology/cytology, antigen testing, or serology consistent with histoplasmosis [3, 14, 20, 21]. Patients with probable histoplasmosis had 1 and 2 above with negative additional testing or did not have additional testing performed. Patients with definite and probable histoplasmosis were included. Patients were excluded if there was low clinical suspicion of probable histoplasmosis diagnosis or another diagnosis was made.

CT findings considered consistent with mediastinal lymphadenopathy/mediastinal granuloma were calcified mass or lymph node conglomeration occasionally with necrosis. CT findings that were considered diagnostic of fibrosing mediastinitis were a well-defined, heterogeneous diffusely infiltrative mass without calcification involving multiple areas of the mediastinum. In both, the lymph node regions of the chest involved were interlobar, hilar, paratracheal, pre-paratracheal, retrotracheal, precarinal, subcarinal, and retrocardiac. Both can be present in a patient causing overlap of CT chest findings [18, 22–24].

Positive serology was defined as complement fixation antibody titers ≥ 1:16 to yeast or mycelial antigens in the blood or the presence of H and/or M precipitin bands by immunodiffusion. Antigen testing was performed on blood and urine. Cytology was performed on body fluids such as bronchoalveolar lavage specimens, pleural fluid, or pericardial fluid and demonstrated yeast-like structures characteristic of Histoplasma capsulatum. Histopathology was performed on mediastinal masses or lung tissue and demonstrated necrotic or fibrotic tissue, or necrotizing granulomas with or without characteristic yeast forms [3, 14, 21, 25]. EIA testing is not done at our institution.

### Data Collection

Symptoms were recorded at the initial patient presentation that led to diagnosis and included chest pain, cough, dyspnea, congestion, syncope, fatigue, hemoptysis, dysphagia, and signs of superior vena cava (SVC) syndrome (swelling in the neck, face, and upper limbs secondary to compression of the SVC preventing blood leaving the upper body). Imaging studies (chest radiographs and computed tomography (CT)), electrocardiograms, and echocardiograms from all encounters the patient had for histoplasmosis during the study time frame were reviewed and results recorded. Interventions such as biopsy and/or surgical resection, pericardiocentesis, pericardial window, and diagnostic or interventional catheterization were documented.

### Cardiovascular Complications

Cardiovascular complications were defined as pericarditis with and without pericardial effusion, cardiac compression, or vascular obstruction involving the pulmonary artery, aorta, inferior vena cava (IVC), superior vena cava (SVC), or pulmonary veins. These conditions were considered secondary to mediastinal granuloma, lymphadenitis, or fibrosing mediastinitis—recognized sequelae of histoplasmosis and were diagnosed based on radiologist interpretation of chest CT [18, 22–24]. Patients without cardiovascular complications were included to provide comparison of demographics and clinical presentation to patients with cardiovascular complications. However, description of complications and interventions was only described for patients with cardiovascular complications.

### Cardiovascular Interventions

Patients with pericardial effusion underwent pericardiocentesis or pericardial window if there were clinical findings concerning for tamponade or if treatment with anti-inflammatory medication did not resolve the effusion. Cardiac catheter intervention was guided by the 2011 guidelines [26]. Patients underwent cardiac catheter intervention of pulmonary arteries if they had significant stenosis as defined as > 20 to 30 mm Hg across the stenosis area or elevation of the right ventricle or proximal main pulmonary artery pressure greater than one half of systemic pressure. A stent was placed if balloon angioplasty did not achieve adequate results. Frequently, only one pulmonary artery was stenosed causing redistribution of blood flow, underestimating the true level of obstruction. Therefore, pulmonary arteries intervention was also performed if the caliber of the vessel was significantly diminished on angiogram or CT. Patients underwent cardiac catheter intervention of the pulmonary veins if the gradient from the vein to the left atrium was > 4mmHg or there was delayed return of contrast to the left atrium during catheter angiogram compared to unobstructed veins. Pulmonary veins were intervened upon if the caliber of the vessel was significantly diminished on angiogram or CT. A stent was placed if balloon angioplasty did not achieve adequate result. Patients underwent cardiac catheter intervention of the SVC if there were clinical concerns for SVC syndrome or if there was radiographic or angiographic evidence of distension of the veins proximal to the obstruction or collateral venous vessels around the obstruction. Patients were referred for surgery if there was compression of structures that could not be relieved via cardiac catheterization, for example atrial compression. Additionally, patients were referred to surgery if additional non-cardiac structures necessitated surgical intervention.

### Statistical Analysis

This is a descriptive study. Continuous variables were not normally distributed and reported as medians. The variables were analyzed using two sample T-test with a p value < 0.05 deemed statistically significant. Categorical variables were summarized using frequencies and percentages. The variables were analyzed using chi square with a p value < 0.05 deemed statistically significant. Multivariate logistic regression analyses were conducted to estimate odds ratios and 95% confidence intervals. Patient symptoms were variables were entered into a multivariate logistic regression model to identify independent predictors of the cardiovascular complications. Adjusted ORs and 95% CIs were reported. All analyses were performed using IBM SPSS Statistics Version 25.

## Results

### Patient Demographics

A total of 160 children were diagnosed with definite or probable histoplasmosis during the study period, with 75 (47%) were female. The median age at diagnosis with histoplasmosis was 12 years. Among these 160 children, 65 (41%) exhibited one or more cardiovascular complications during the time frame of the study, including 27 females (42% of those with complications), the median age at diagnosis of with histoplasmosis was 12 years. No significant differences in sex distribution between patients with and without cardiovascular complications (53% male versus 47% female; χ^2^ = 0.808, *p* = 0.368) (Table 1).​

### Clinical Presentation

151 patients presented to our emergency department or another emergency department for symptoms listed in Table 1. They were then admitted from our emergency department or transferred to our institution for further work up. 5 patients were transferred from St. Jude after cancer was ruled out or due to critical ill status secondary to disseminated histoplasmosis. For 4 patients, the first encounter was cardiology clinic after referral for chest pain or murmur then later admitted for work up and intervention. Patients without cardiac involvement, during the time frame of the study, more commonly presented with fever, cough, hemoptysis, dysphagia, and congestion at initial encounter. However, only cough (70% versus 30%; *χ*^*2*^ = 12.19, *p* = 0.00048) and hemoptysis (100% versus 0%; *χ*^*2*^ = 4.61, *p* = 0.03177) were associated with non-cardiac involvement. In contrast, those with cardiac manifestations, during the time frame of the study, were more likely to experience chest pain, dyspnea, syncope, and fatigue at initial encounter. However, only chest pain was associated with cardiac involvement (59% versus 41%; *χ*^*2*^ = 4.23, *p* = 0.03961). Among 73 children presenting with chest pain, 43 were subsequently found to have cardiovascular sequelae (OR 4.9; 95%CI 1.4–16.8; *P* = 0.01). Hemoptysis was attributed to pulmonary involvement as no patient with initial presentation of hemoptysis had developed cardiac complications. A single patient presented with facial swelling indicative SVC syndrome. The physical examination finding of muffled heart sounds was associated with cardiac involvement (100% versus 0%; *χ*^*2*^ = 14.02, *p* = 0.00018), whereas, the presence of a murmur or pericardial friction rub did not reliably predict such complications (Table 1).

**Table 1:** Demographics of patients diagnosed with histoplasmosis, with and without cardiac involvement. Presenting symptoms and cardiac exam findings of patients at time of initial presentation that led to diagnosis. Some patients had multiple symptoms

	Patients WITH cardiac complications from Histoplasmosis	Patients WITHOUT cardiac complications from Histoplasmosis	Total Patients	*P*-Value
Patients	65	95	160	
Male	38 (58%)	47 (49%)	85 (53%)	χ^2^ = 0.588, *p* = 0.443
Female	27 (42%)	48 (51%)	75 (47%)	χ^2^ = 0.667, *p* = 0.414
Median age at presentation	12 years	11 years	12 years	
Presenting Symptoms				
Chest Pain	43 (59%)	30 (41%)	73	χ^2^ = 4.23, *p* = 0.03961
Cough	25 (30%)	59 (70%)	84	χ^2^ = 12.19, *p* = 0.00048
Dyspnea	27 (55%)	22 (45%)	49	χ^2^ = 1.02, *p* = 0.31867
Fever	37 (42%)	51 (58%)	88	χ^2^ = 0.99, *p* = 0.31215
Congestion	15 (39%)	23 (61%)	38	χ^2^ = 0.72, *p* = 0.39750
Syncope	2 (67%)	1 (33%)	3	χ^2^ = 0.01, *p* = 0.92557
Fatigue	17 (53%)	15 (47%)	32	χ^2^ = 0.25, *p* = 0.62226
Hemoptysis	0 (0%)	7 (100%)	7	χ^2^ = 4.61, *p* = 0.03177
Dysphagia	3 (43%)	4 (58%)	7	χ^2^ = 0.0, *p* = 1
SVC Syndrome	1 (100%)	0 (0%)	1	χ^2^ = 0.0, *p* = 0.95709
Presenting Clinical Signs				
Murmur	6 (60%)	4 (40%)	10	χ^2^ = 0.24, *p* = 0.62226
Pericardial Friction Rub	4 (100%)	0 (0%)	4	χ^2^ = 2.62, *p* = 0.10576
Muffled Heart Sounds	14 (100%)	0 (0%)	14	χ^2^ = 14.02, *p* = 0.00018

### Cardiovascular Manifestations and Interventions

**Fig. 1:**
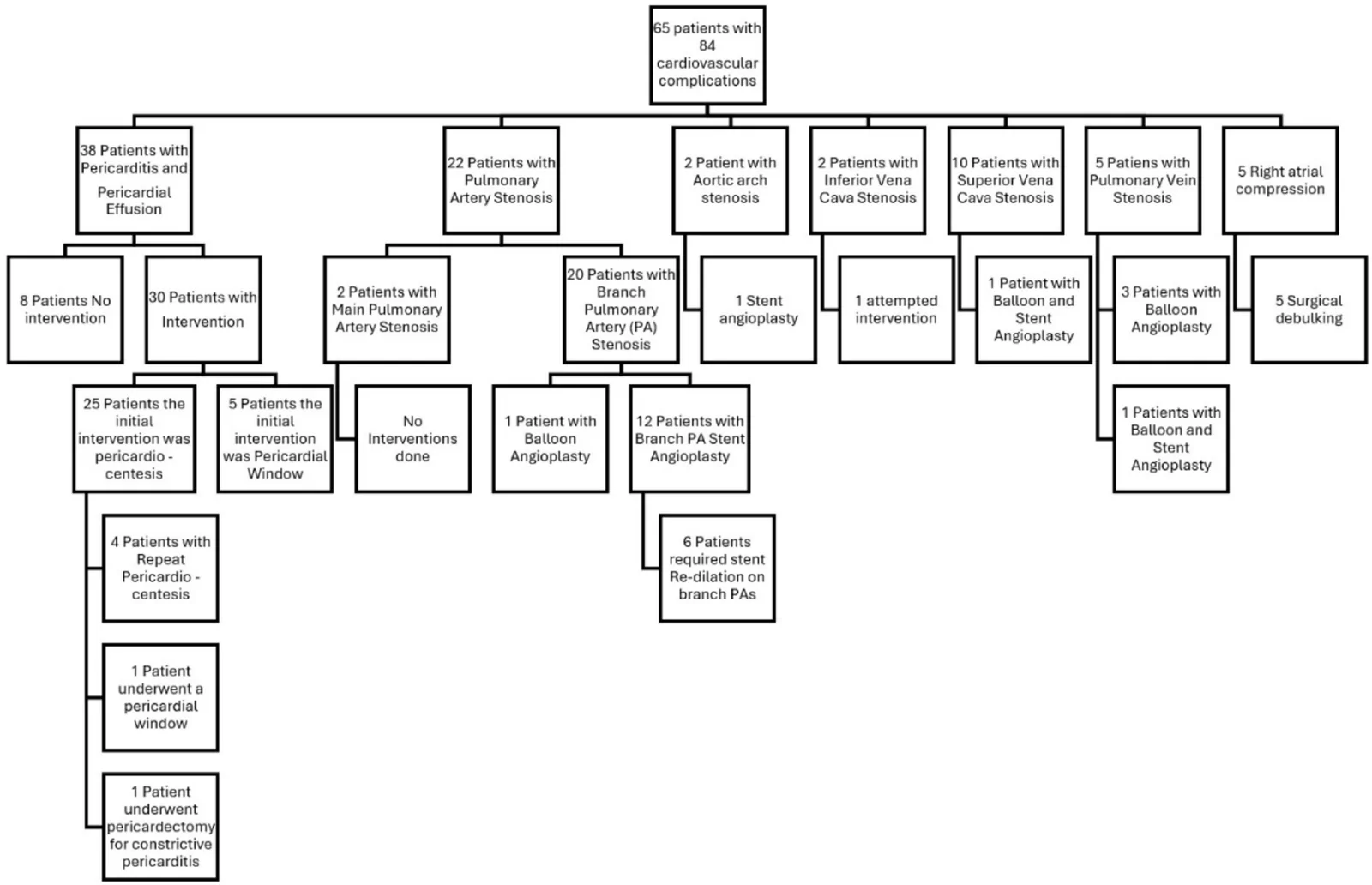
Cardiovascular complications secondary to mediastinal manifestations of histoplasmosis in children and the corresponding interventions performed

Figure 1 summarizes the cardiac manifestations, and the interventions performed. Some patients had multiple cardiac manifestations during the timeframe of the study. These manifestations were present at time of histoplasmosis diagnosis or were diagnosed at a subsequent encounter.

Pericarditis with pericardial effusion was the most prevalent cardiovascular complication (Fig. 1), occurring in 38 of the 65 patients (58%). Thirty patients with pericardial effusions required intervention via pericardiocentesis or pericardial window. For instance, one patient presented with chest pain, shortness of breath, and fever; imaging revealed cardiomegaly and a large pericardial effusion, necessitating drainage of 250 mL of serosanguinous fluid. The mean volume of fluid drained was 506 mL (with a standard deviation of 315 ml). Twenty of 30 drained effusions (67%) were serosanguinous or hemorrhagic. Four patients required repeat pericardiocentesis due to re-accumulation, with intervals averaging three days between procedures. Subsequent interventions included pericardial window in one patient (2 days after initial procedure) and pericardiectomy in another (178 days after the initial procedure).​ The pericardiectomy was performed secondary to severe pericardial fibrosis and signs of constrictive pericarditis.

**Fig. 2:**
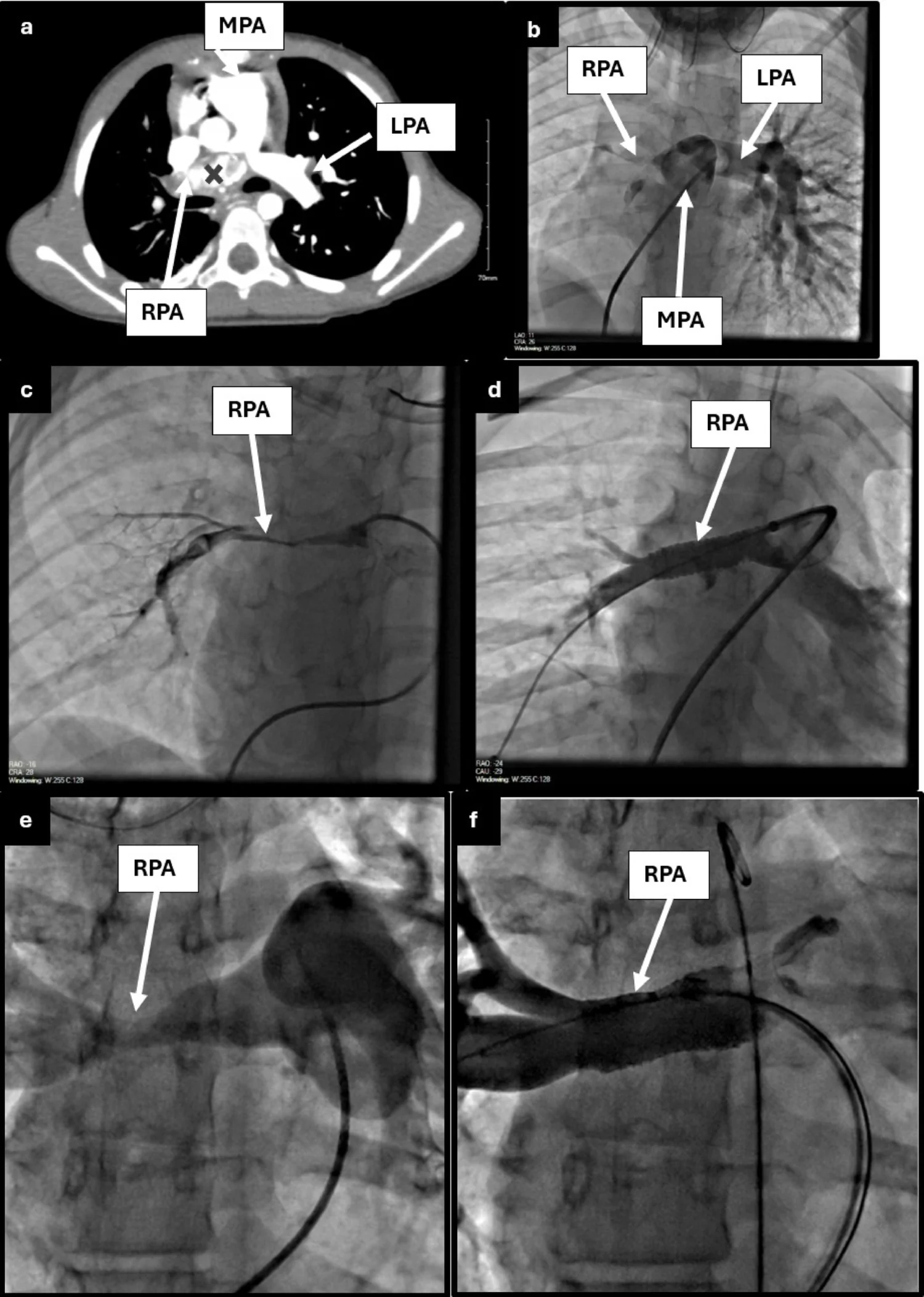
Imaging of RPA compression and intervention. 6-year-old male (**a**) chest CT angiogram demonstrates extensive calcified middle mediastinal lymph nodes (X) causing significant compression of the RPA. **b** and **c** Angiograms in the cardiac catheterization laboratory reveal preferential flow into the LPA and long-segment stenosis of the RPA. **d** Post-stent placement angiogram of the RPA demonstrates interval increase in luminal diameter and improved flow. 13-year-old female (**e**) angiogram with moderate to severe RPA stenosis prior to intervention and (**f**) Post-stent implantation. MPA = main pulmonary artery; LPA = left pulmonary artery; RPA = right pulmonary artery

**Fig. 3:**
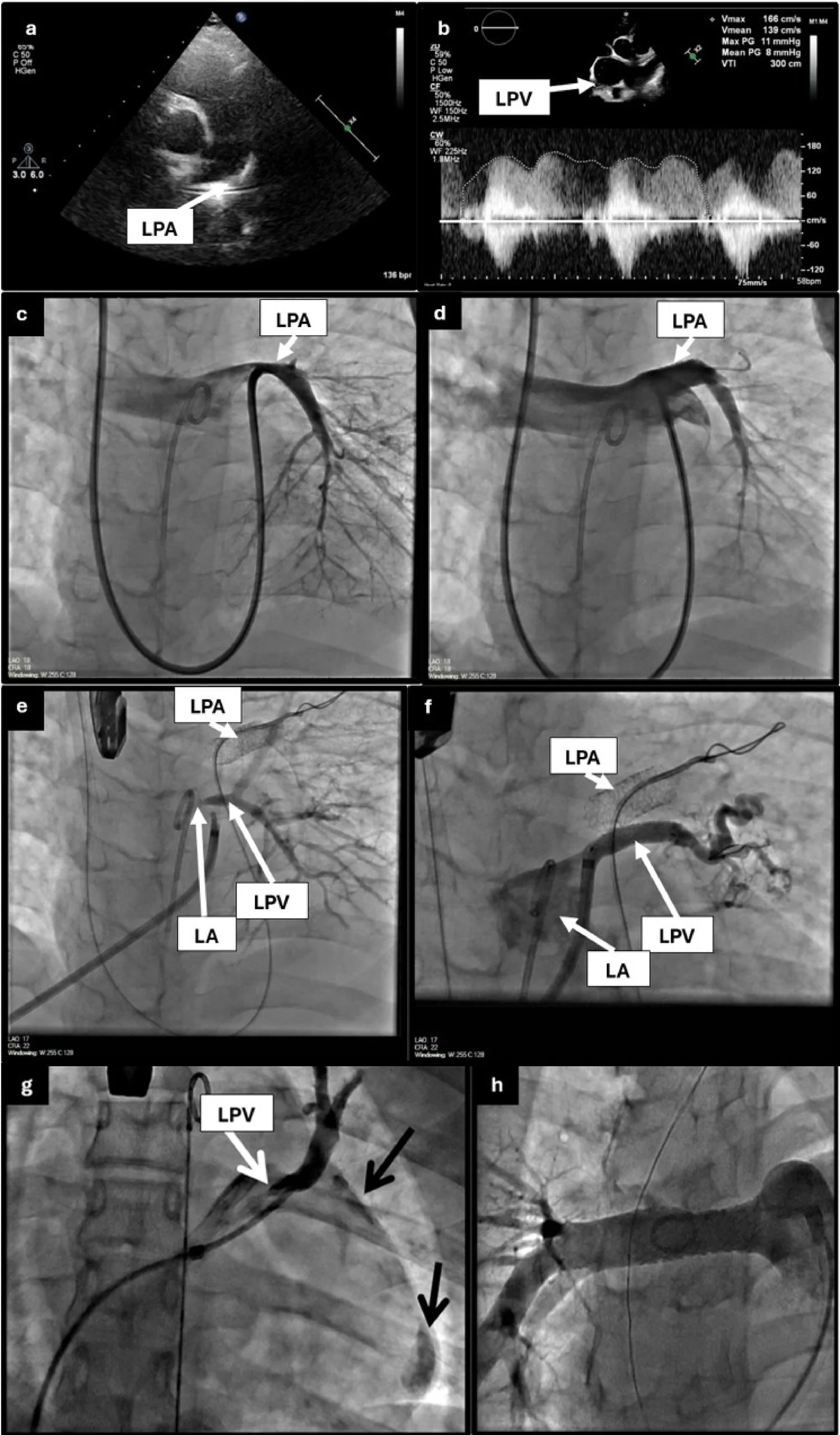
Multimodal imaging and intervention for pulmonary artery and pulmonary vein stenosis. 15-year-old male with fibrosing mediastinitis (**a**) Parasternal short-axis echocardiographic view reveals severe proximal LPA stenosis and (**b**) significant LPV stenosis with a gradient of 8 mmHg. The patient was referred for cardiac catheterization where (**c**) selective angiogram in the LPA reveals washback into the RPA, LPA measures 5 mm at its narrowest. **d** Angiogram following stent placement in the LPA with increase in vessel diameter. **e** Pulmonary artery wedge angiogram in the left upper pulmonary artery branch demonstrates near atresia of the LPV. **f** After stent placement, there is unobstructed contrast flow into the LA. 12-year-old male with fibrosing mediastinitis who subsequently required pericardiectomy for signs of constrictive pericarditis. **g** Pericardial calcifications (Black arrows) seen on angiogram of left upper pulmonary vein stenosis. **h** The pulmonary vein stenosis responded to balloon angioplasty alone. The RPA required stent implantation. LPA = left pulmonary artery; RPA = right pulmonary artery; LA = left atrium; LPV = left pulmonary vein

Additional cardiac manifestations seen in pediatric patients are related to obstruction or compression. Pulmonary artery stenosis, pulmonary vein stenosis, aortic stenosis, SVC stenosis, and IVC stenosis were diagnosed at time of histoplasmosis diagnosis or at a subsequent encounter. Pulmonary artery stenosis was the second most common complication, identified in 22 patients (34%). While two cases of main pulmonary artery stenosis required no intervention, 13 of the 20 patients with branch pulmonary artery stenosis underwent procedures: 12 received stent angioplasty with or without attempted initial balloon dilatation, and one underwent successful balloon angioplasty alone. A total of 17 stents were used in the pulmonary arteries. 12 were ev3 IntraStent Max LD, 4 were express LD, and one was a Palmez Blue stent. The average peak to peak gradient of the affected branch pulmonary artery to the main pulmonary artery prior to intervention was 18.2 mmHg and after intervention was 3.4 mmHg. On average the right ventricle pressure was 47% of left ventricle pressure prior to intervention and 32% after intervention. Four of these patients required repeat intervention on the pulmonary artery stents within 3 months to 5 years after stent placement, 2 had ev3 IntraStent Max LD stents and 2 had express LD stents. The primary indications for reintervention were echocardiographic evidence of elevated right ventricular pressures, increased gradients, or decreased diameter necessitating further angioplasty or stent dilatations to accommodate patient growth or continued mediastinal disease; however, only one patient was symptomatic at the time of re-intervention.​ Two patients also received stent implantations in the contralateral branch pulmonary artery 12 months and 32 months after the initial stent (Figs. 2 and 3). 7 of the 22 patients with main or branch pulmonary artery stenosis had a pericardial effusion 1 to 5 years prior.

**Fig. 4:**
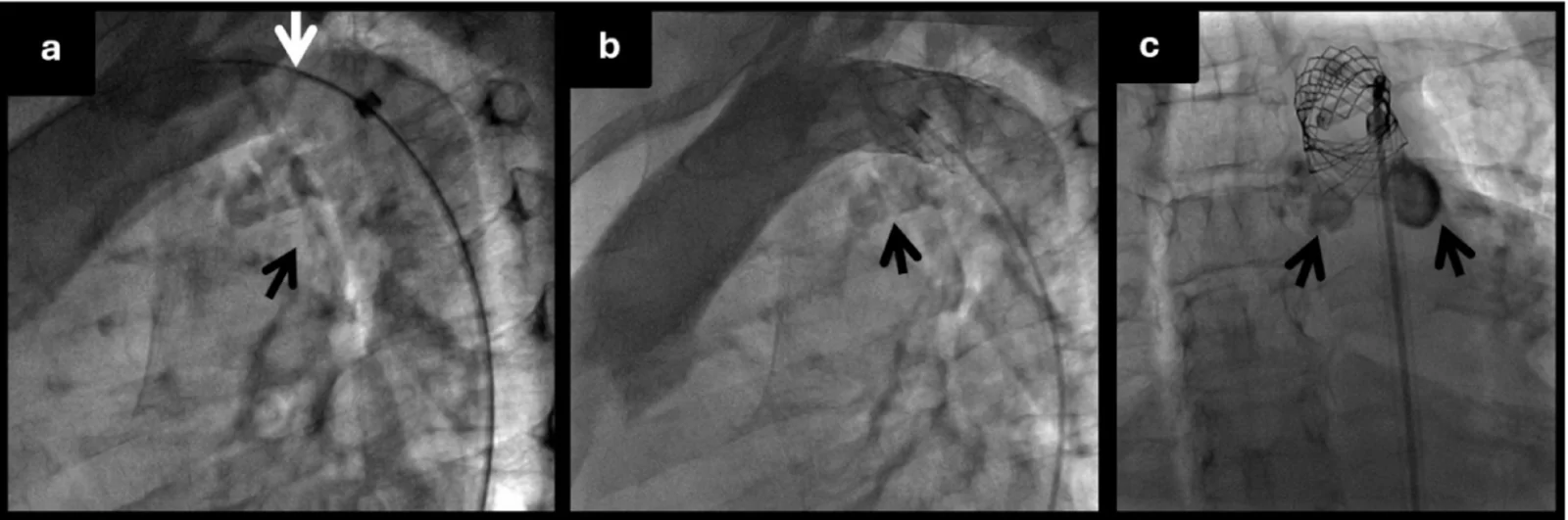
Angiographic images of a 16-year-old male with aortic coarctation secondary to large perivascular calcified lymph node. **a** Lateral projection of the aortic arch and descending aorta demonstrates a discrete area of stenosis (white arrow) adjacent to two large perivascular calcified lymph nodes (black arrow). **b** and **c** Angiography following balloon angioplasty and stent placement shows near resolution of the obstruction

Pulmonary vein stenosis was observed in five patients, with three requiring balloon angioplasty and one necessitating stenting. The latter case involved extensive left hilar disease, leading to four catheterization procedures for stenting of the left upper and lower pulmonary veins as well as the left pulmonary artery (Fig. 3). Initially, Medtronic Resolute Onyx stents were placed in left upper and lower pulmonary veins and later these veins were re-stented with Cook Formula stents, left upper pulmonary vein 7 months later and left lower pulmonary vein 2 years later. Aortic involvement was rare; one patient exhibited compression of the descending aorta compression without significant echocardiographic gradient, thus not warranting intervention.​ Another patient had significant distal aortic arch stenosis secondary to perivascular calcified lymphadenopathy requiring stent implantation (Fig. 4).

**Fig. 5:**
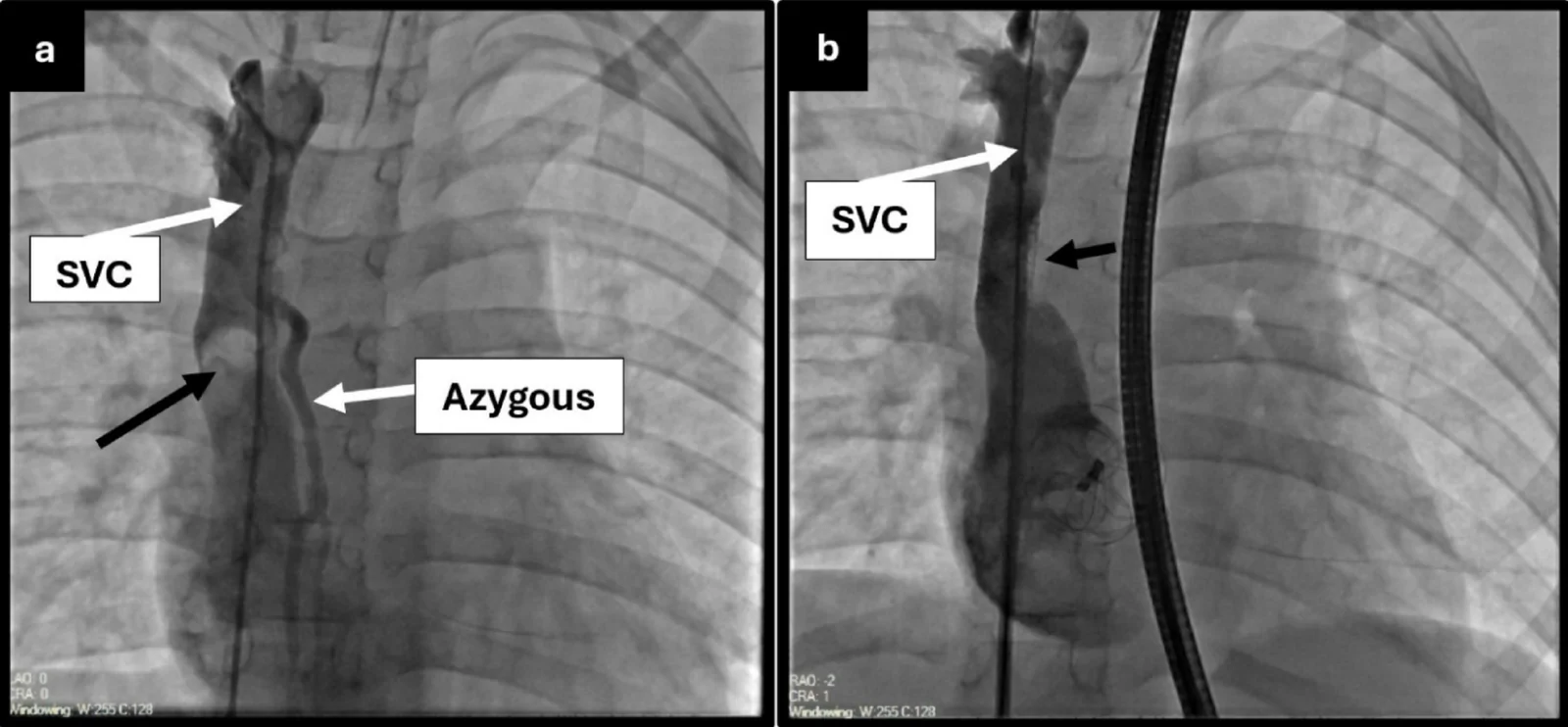
7-year-old female with superior vena cava syndrome secondary to severe extrinsic mass effect from the necrotic mediastinal lymphadenopathy. **a** Frontal projection angiography shows filling defect of the SVC near the right atrium from the lymphadenopathy (black arrow). There is decompression of blood flow into the azygous vein due to high pressure in the SVC. After stent was placed in SVC (black arrow), **b** the filling defect is no longer seen and there is no decompression of blood flow into the azygous vein. Of note patient’s patent foramen ovale was also occluded prior to the procedure and the device is seen. SVC = superior vena cava

SVC compression was noted in 10 patients via CT angiography, though only 1 presented with clinical SVC syndrome, characterized by dilated neck veins and periorbital edema. This patient had near-complete SVC lumen obliteration due to necrotic mediastinal lymphadenopathy and underwent successful stenting with an Express stent (Fig. 5). Inferior vena cava (IVC) involvement was identified in 2 patients with calcified lymphadenopathy extending out of the mediastinum: 1 with complete occlusion, however recanalization was unsuccessful; 1 with compression not severe enough to require intervention.

In total, 14 patients underwent catheter-based stenting procedures with a total of 22 stents placed (17 in pulmonary artery branches, 4 in pulmonary veins, and 1 in the SVC). No mortality from catheterization intervention was observed in our cohort. Only 2 patients experienced procedural complications: one patient who needed bilateral pulmonary artery stents secondary to fibrosing mediastinitis developed multiple thrombi during the procedure, necessitating the administration of transcatheter tissue plasminogen activator (TPA), and the other had a pseudoaneurysm of the left pulmonary artery at the site of angioplasty.

Mass resection was performed in 5 cases with cardiac involvement, the surgical indication was right atrial compression from mass effect. There was no surgical mortality reported.

In addition to catheter interventions, patients in the study were treated with antifungals and anti-inflammatory medication. In immunocompetent hosts, cardiac complications are due to pericarditis and extrinsic compression from mediastinal granuloma or fibrosing mediastinitis. Treatment is anti-inflammatories (NSAIDs and/or steroids) for patients with pericarditis with and without effusion. While antifungals are often given, they are mostly given as a precaution when immunosuppressive anti-inflammatory treatments are given (steroids, rituximab), but are not felt to impact disease from extrinsic compression as there is rare live fungus present (mediastinal granuloma) or none (FM) which requires mechanical treatment. It is possible for a patient with disseminated disease to have a pericardial effusion, in that scenario antifungal treatment is targeting the disseminated fungal infection. Patients who underwent stent implantation did not have any post procedure antithrombotic regimen. Given this is a pediatric population, patient growth in addition to lymph node progression (calcified versus mediastinal granuloma versus fibrosing mediastinitis) contribute to restenosis of previously intervened structures or development of new stenosis. Therefore, all patients followed up in cardiology clinic to monitor for reaccumulation of effusion or stenosis. However, there is variability between patients so follow up was based on physician clinical judgement and patient status, our institution does not have set guidelines.

### Imaging Findings

**Fig. 6:**
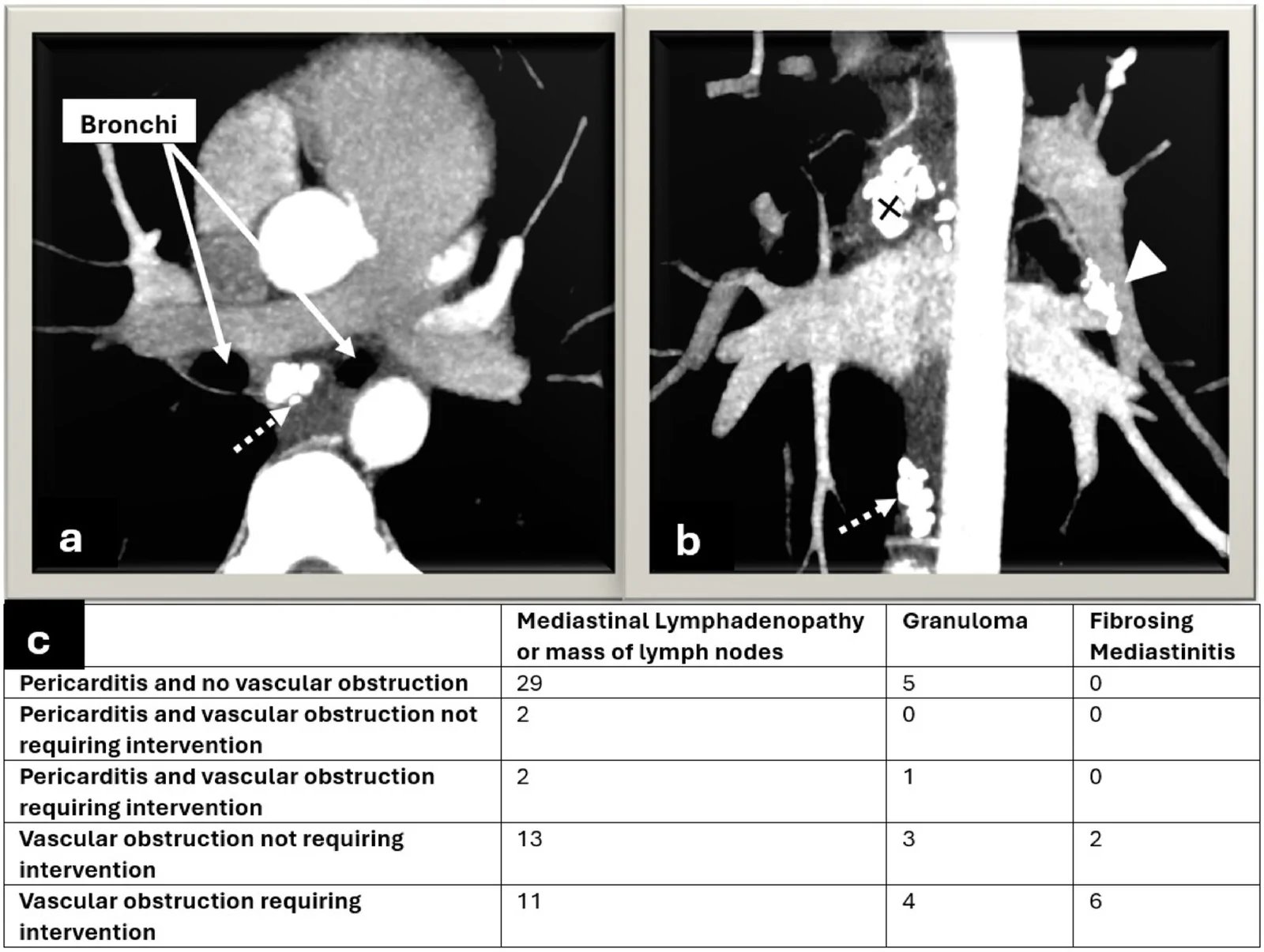
CT findings. CT thorax of a 6-year-old male with fibrosing mediastinitis (**a**) Axial image shows subcarinal calcified lymph nodes (white dashed arrows) located between the left and right mainstem bronchi, with mild compression of the left mainstem bronchus. **b** Frontal projection showing paraesophageal calcified lymph nodes to the right of the descending aorta (black X). The large subcarinal calcified lymph nodes are again visualized, along with a distinct area of calcified left hilar lymph nodes (white arrow head). **c** Summary of the CT chest findings in patients with cardiovascular manifestations of histoplasmosis. CT = computed tomography

CT imaging of patients with cardiovascular complications consistently revealed granulomas and mediastinal lymphadenopathy, involving nodes such as interlobar, hilar, paratracheal, pre-paratracheal, retrotracheal, precarinal, subcarinal, and retrocardiac lymph nodes (Fig. 6). These mediastinal lymph node regions associated with lower risk of lymphoma whereas anterior mediastinal and paratracheal nodes are not often involved [18, 22]. The extent and volume of lymphadenopathy and granulomas correlated with the severity of cardiac structure involvement. While most lymphadenopathy was calcified, eleven of the 65 patients exhibited necrotic features consistent with mediastinal granuloma. All 8 of the 65 patients diagnosed with fibrosing mediastinitis on CT had cardiac involvement, predominantly affecting branch pulmonary arteries and veins, often necessitating catheter-based interventions in 5 of 8 cases (Fig. 6). Patients with fibrosing mediastinitis also had calcified lymphadenopathy and granulomas as these two manifestations can occur in the same patient.

## Discussion 

Histoplasmosis, a subclinical infection in immunocompetent individuals, can manifest or lead to significant cardiovascular complications in the pediatric population, such complications being particularly prevalent in hyper-endemic regions. This retrospective study over 14 years provides a comprehensive analysis of such complications in children.​

Pericarditis with associated pericardial effusion was the most common cardiac manifestation affecting 24% of pediatric patients with histoplasmosis compared to 10–15% of adults [7]. In particular, 58% of children with cardiovascular complications had a pericardial effusion. These patients frequently presented with chest pain, shortness of breath, and signs of tamponade physiology, requiring prompt interventions such as pericardiocentesis or pericardial window procedures. This array of clinical features is similar to findings from another hospital in an endemic region reporting on patients with pericarditis due to histoplasmosis, 66% of whom required pericardiocentesis and most common presenting symptoms were chest pain, shortness of breath, and fever [6]. We hypothesize that granulomatous, subcarinal lymph nodes may erode into pericardial veins, causing hemorrhage into the pericardial space, which leads to an acute on chronic manifestation of the disease process as there is evidence of chronic histoplasmosis lung changes at time of pericardial effusion. The average volume of fluid drained was 506 mL, with the effusions often being sero-sanguineous or hemorrhagic in nature. Serous effusions were rare (16%). Importantly, some patients, especially with serous effusions, required repeat interventions due to fluid re-accumulation, indicating the potential for persistent or recurrent effusions in this cohort.

Additional cardiac complications arose from the compression of cardiac structures and were present at the time of diagnosis of histoplasmosis or developed later. Compression occurred from lymphadenopathy some of which had undergone chronic changes and progressed to mediastinal granulomas or fibrosing mediastinitis which are two distinct complications of histoplasmosis. Differentiating between these two conditions on imaging can be challenging and both can be present in the same patient. While granulomas are typically well-defined and may have an enhanced rim, fibrosing mediastinitis is characterized by a progressive fibrotic reaction involving multiple nodal regions, leading to obliteration of fascial planes. Distinguishing between the two typically requires a biopsy, though this is rarely performed unless patients have severe symptoms. Another distinguishing factor is the natural progression of the obstruction. Several patients had serial CT scans that demonstrated the evolution of fibrosing mediastinitis. Initially, there were single or multiple calcified lymph nodes, which then enlarged and coalesced into masses and as fibrosis progressed, the fascial planes were completely obliterated. Mediastinal granulomas may also enlarge, encroach on nearby structures, or undergo necrosis and involution [27, 28]. In this study, all patients diagnosed with fibrosing mediastinitis exhibited cardiac involvement, with many requiring interventional procedures. If these interventions were not performed promptly and aggressively, the fibrosis and calcification can become resistant to any form of therapeutic attempts and major vessel permanently obstructed.

If the mediastinal disease occurs in the hilum of the lungs, there is external compression of branch pulmonary arteries. Pulmonary artery stenosis was the second most common cardiovascular complication, affecting 34% of patients. Due to significant mediastinal fibrosis or external compression of the branch pulmonary arteries by calcific lymphadenopathy, balloon angioplasty was largely ineffective, making stent angioplasty the preferred treatment (Figs. 2 and 3). The degree of fibrosis and calcification in these cases is comparable to the challenges encountered when treating a calcified homograft, a common problem in repaired Tetralogy of Fallot patients. Furthermore, recoil of the stent when trying to place it in an area of stenosis was a frequent observation. Both challenges lead to the use of multiple stents or stents with higher radial strength. Stenoses of the superior vena cava (SVC) was approached in a similar manner to documented techniques used to relieve thrombotic obstruction of the SVC in infants with post-operative complications (Fig. 5) [29]. Stenosis of the inferior vena cava (IVC) was observed, with intervention reserved for symptomatic patients or those with significant hemodynamic compromise. Although less common, pulmonary vein stenosis also required similar interventions in select cases (Fig. 3). In all, 14 patients had stent angioplasty in the 14 years of our study compared to the adult study by Alders where 40 patients had stent angioplasty in 12 years [30].

There is high mortality when intervening surgically to treat these vessel stenoses due to obliteration of the facial planes by fibrotic reaction. This study underscores the feasibility and efficacy of percutaneous interventions in managing vascular complications arising from histoplasmosis-related mediastinal involvement which is our preferred approach compared to surgery in adults [31–34]. Catheterization also has a role in further delineation of anatomy and obstruction, for example pulmonary vein stenosis may be congenital and not related to histoplasmosis [34]. No mortalities were observed in our cohort. Only two patients experienced procedural complications: one patient who needed bilateral pulmonary artery stents secondary to fibrosing mediastinitis developed multiple thrombi during the procedure, necessitating the administration of TPA, and the other had a pseudoaneurysm of the left pulmonary artery at the site of angioplasty.

Adult patients have additional cardiovascular manifestations of histoplasmosis including endocarditis affecting native and prosthetic valves, mycotic aortic aneurysm, aortic graft infection, and myocarditis [8–11].​ These complications are rare and were not observed in our pediatric cohort.

This study is limited by the subclinical nature of histoplasmosis in an immunocompetent host therefore the true number of infected patients is unknown as well as asymptomatic cardiac manifestations. Diagnosis is challenging given the gold standard of isolating the fungus in culture takes several weeks. Other immunologic tests have been developed, however there is cross reactivity with other fungi and test results can be normal in a patient without acute infection. Therefore, some patients are diagnosed as probable histoplasmosis. Longitudinal follow up is difficult given inconsistent follow up amongst patients who are asymptomatic despite ongoing obstructive process. Therefore, the true number of patients with recurrent or new obstruction may be underestimated.

## Conclusion

In regions hyperendemic for histoplasmosis, pediatric patients are at risk for serious cardiovascular complications, notably pericarditis with pericardial effusion and vascular stenoses due to mediastinal involvement. Prompt recognition and intervention are crucial to mitigate morbidity from cardiac complications. This study highlights the importance of considering histoplasmosis in the differential diagnosis of pediatric patients presenting with cardiovascular symptoms in endemic areas and supports the role of percutaneous interventions in managing associated complications.

## Supplementary Information

Below is the link to the electronic supplementary material.

Supplementary Material 1

## Data Availability

No datasets were generated or analysed during the current study.
