# Supplementary material for: Cardiovascular Manifestations and Interventions in Children Living in a Histoplasmosis Endemic Region
Source: Pediatr Cardiol. 2025 Dec 2;47(8):3362–73. doi: 10.1007/s00246-025-04123-w (PMC13687199; doi:10.1007/s00246-025-04123-w)
Supplement: Supplementary file 1 [file 246_2025_4123_MOESM1_ESM.docx]

**Supplemental Materials**

ICD-10 (and ICD-9) Codes used:

B39.9 (115.90) Histoplasmosis and Histoplasmosis

B39.0 (115.05) Acute pulmonary histoplasmosis capsulati

B39.1 (115.05) Chronic pulmonary histoplasmosis capsulati

B39.2 (115.05) Pulmonary histoplasmosis capsulati, unspecified

B39.3 (115.09) Disseminated histoplasmosis capsulati

B39.4 (115.00) Histoplasmosis capsulati, unspecified

B39.5 (115.10) Histoplasmosis duboisii

B39.9 (115.90 and 115.93 and 115.99) Histoplasmosis, unspecified
